# Fluoxetine Enhances Synaptic Vesicle Trafficking and Energy Metabolism in the Hippocampus of Socially Isolated Rats

**DOI:** 10.3390/ijms232315351

**Published:** 2022-12-05

**Authors:** Dragana Filipović, Victor Costina, Peter Findeisen, Dragos Inta

**Affiliations:** 1 Department of Molecular Biology and Endocrinology, “VINČA”, Institute of Nuclear Sciences—National Institute of thе Republic of Serbia, University of Belgrade, 11000 Belgrade, Serbia; 2 Institute for Clinical Chemistry, Medical Faculty Mannheim of the University of Heidelberg, University Hospital Mannheim, 68159 Mannhem, Germany; 3 Department for Community Health Faculty of Natural Sciences, Medicine University of Fribourg, 1700 Fribourg, Switzerland; 4 Department of Biomedicine, University of Basel, 4052 Basel, Switzerland

**Keywords:** fluoxetine, rat hippocampus, proteomics, synaptosomes, synaptic mitochondria

## Abstract

Chronic social isolation (CSIS)–induced alternation in synaptic and mitochondrial function of specific brain regions is associated with major depressive disorder (MDD). Despite the wide number of available medications, treating MDD remains an important challenge. Although fluoxetine (Flx) is the most frequently prescribed antidepressant, its mode of action is still unknown. To delineate affected molecular pathways of depressive-like behavior and identify potential targets upon Flx treatment, we performed a comparative proteomic analysis of hippocampal purified synaptic terminals (synaptosomes) of rats exposed to six weeks of CSIS, an animal model of depression, and/or followed by Flx treatment (lasting three weeks of six-week CSIS) to explore synaptic protein profile changes. Results showed that Flx in controls mainly induced decreased expression of proteins involved in energy metabolism and the redox system. CSIS led to increased expression of proteins that mainly participate in Ca^2+^/calmodulin-dependent protein kinase II (Camk2)-related neurotransmission, vesicle transport, and ubiquitination. Flx treatment of CSIS rats predominantly increased expression of proteins involved in synaptic vesicle trafficking (exocytosis and endocytosis), and energy metabolism (glycolytic and mitochondrial respiration). Overall, these Flx-regulated changes in synaptic and mitochondrial proteins of CSIS rats might be critical targets for new therapeutic development for the treatment of MDD.

## 1. Introduction

Chronic psychosocial stress is one risk factor for developing major depressive disorder (MDD). Its etiology is not yet fully elucidated, but several reports indicate synaptic and mitochondrial dysfunctions [1,2]. Hence, impaired neurotransmission of monoaminergic pathways, including serotonin, has been reported in MDD [3,4]. Clinical and preclinical studies showed reduced inhibitory neurotransmitter γ-aminobutyric acid (GABA) and glutamic acid decarboxylase 67 (GAD67) levels in the brain, including decreased protein expression of parvalbumin as GABAergic interneuron [5,6]. A study in a chronic mild stress-based animal model of depression showed impairment of glutamate/GABA presynaptic release, brain-derived neurotrophic factor (BDNF) mRNA trafficking in dendrites, and reduced length of apical dendrites in CA3 pyramidal neurons of the hippocampus [7]. In addition, various models of chronic stress exposure in rodents have indicated a reduction in neurogenesis in the hippocampal dentate gyrus (DG) [8]. Moreover, mitochondrial dysfunction that results in decreased electron transport chain (ETC) and ATP production, impaired bioenergetics capacity, and increased oxidative stress and apoptosis, appears to be contributing a factor in the etiopathogenesis of MDD [1,9,10]. Experimental data suggest a critical role of synaptic mitochondria in synaptic vesicle pools function and organization and neurotransmitter release during intense neuronal activity [11]. Therefore, intensive research on MDD is focused on finding molecular mechanisms in brain synapses.

For the treatment of MDD, fluoxetine (Flx), as a selective serotonin reuptake inhibitor (SSRI), is the most frequently prescribed antidepressant [12] which inhibits serotonin reuptake by blocking serotonin transporters, resulting in increased serotonin synaptic levels. Independent of serotonin reuptake blockade, Flx exerts therapeutic effects by promoting neuronal plasticity through its direct binding to the tropomyosin-related receptor kinase B (TrkB), which is a cholesterol-sensitive receptor [13] and receptor for BDNF [14]. Its administration in mice induces changes in synaptic plasticity in the hippocampus and functional dynamics associated with changes in hippocampal-dependent behavior and expression of synaptic proteins that regulate neurotransmitter turnover and release [15]. Notably, Flx interacts with mitochondria, altering their function by modulating the protein expression and activity of respiratory chain components and enzymes of the citrate cycle (TCA) [16,17]. Moreover, Flx rapidly blocks serotonin reuptake, but the onset of the therapeutic response is delayed by several weeks [18], indicating that its chronic treatment requires optimization. Hence, the synaptic dysfunctions observed in clinical and preclinical studies might represent a novel therapeutic target.

In our study, we used chronic social isolation (CSIS) as a validated rat model of depression [19,20,21,22]. We previously demonstrated that rats exposed to CSIS showed anhedonia, a key symptom of MDD (reduction in sucrose preference test), behavioral despair (increased immobility time in the forced swim test), and anxiety symptoms (increased the burying of marbles) [23,24,25].

To delineate affected molecular pathways of depressive-like behavior and to identify potential molecular markers/targets upon Flx treatment, we carried out a nonhypothesis-driven comparative proteomic analysis of hippocampal purified synaptic terminals (synaptosomes) of rats exposed to six weeks of CSIS and/or followed by Flx treatment (lasting three weeks of six-week CSIS) to explore synaptic protein profile changes. Synaptosomes were used since they represent isolated synaptic terminals from neurons that contain mitochondria, synaptic vesicles, and postsynaptic density [26]. In this study, we chose the hippocampus, a brain structure that shows profound alterations in MDD, particularly of altered neural plasticity in response to stress, which plays a role in the onset and development of MDD [27,28]. Moreover, this brain region represents a key target of serotonergic afferents [29]. So far, no studies have examined synaptoproteome in the hippocampus of adult male CSIS rats and/or followed by Flx treatment. Western blot analysis was carried out to validate the proteins representative of altered signal pathways. The identified altered molecular pathways and synaptosomal proteins might be used as potential molecular markers/targets in studying the hippocampal synaptic function and further clarifying the mechanism of MDD and antidepressant action.

## 2. Results

### 2.1. Differential Proteomics Analysis

The lists of commonly identified proteins in the synaptosomal fractions of the rat hippocampus with fold changes are reported in Table 1, Table 2 and Table 3. Under the mentioned terms, Controls treated with Flx resulted in 20 downregulated proteins (Table 1). CSIS compared to Controls, increased the expression of eight proteins (Table 2). Differential proteomic results of Flx-treated CSIS and vehicle-treated CSIS rats showed 85 upregulated and 6 downregulated proteins (Table 3). 

We identified deregulated proteins between CSIS + Flx vs. CSIS group, CSIS vs. Controls (Supplementary Table S1), and CSIS + Flx vs. CSIS and Controls + Flx groups (Supplementary Table S2). Thus, two synaptosomal proteins were downregulated, while one protein had a similar abundance following CSIS + Flx vs. CSIS (Supplementary Table S1). By comparing the list of proteins with altered expression following Flx treatment in CSIS and Controls, we identified four proteins downregulated by Flx in Controls and upregulated by Flx in CSIS (Supplementary Table S2).

### 2.2. Analysis of Protein–Protein Network Interaction by STRING

All synaptosomal differentially expressed proteins were uploaded into the STRING 11.0 software to describe protein interactions and the most frequent biological, molecular, and KEGG pathway assignments. In Controls treated with Flx vs. Control rats, a significant interaction among downregulated proteins was revealed with enrichment *p* < 1 × 10^−16^. Flx mainly induced downregulation of the proteins related to malate metabolic process, tricarboxylic acid cycle (TCA) and NADH metabolic process. The malate dehydrogenase activity and anion binding as molecular functions were found. Significant enrichment KEGG pathways were pyruvate metabolism and TCA cycle. CSIS induced the changes in protein expression with no significant interactions observed within upregulated synaptosomal proteins with no specific biological process and molecular function. In Flx-treated CSIS compared with CSIS rats, a significant interaction among upregulated synaptosomal proteins was found, with enrichment *p* < 1 × 10^−16^. In the aspect of biological process, these proteins mainly take part in the regulation of synaptic vesicle recycling and ATP biosynthetic process and transport, specifically those involved in protein transport (intracellular and vesicle-mediated transport in synapse). In the aspect of molecular function, they possess anion binding with synaptic vesicle cycle as a KEGG pathway. 

**Figure 1:**
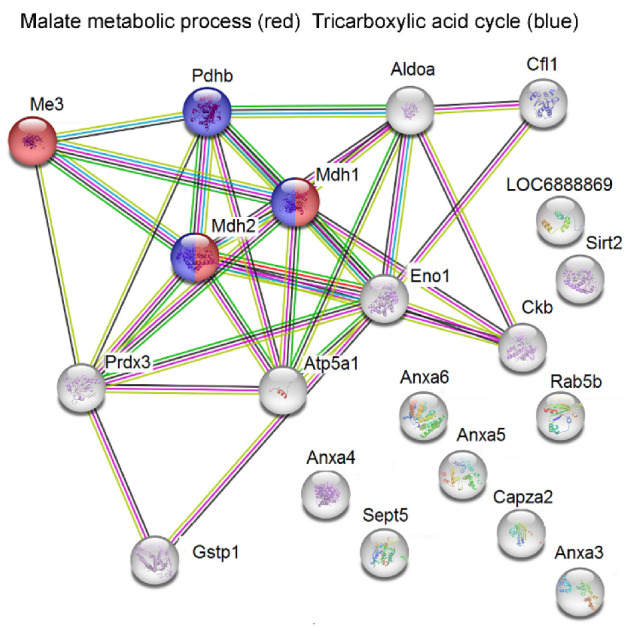
STRING-based detection of modified biological pathways among synaptosomal differentially downregulated proteins following Flx treatment in Control rats; in red, proteins involved in regulation of malate metabolic process; in blue, proteins involved in tricarboxylic acid cycle; Mdh2–malate dehydrogenase, mitochondrial; Mdh1–malate dehydrogenase, cytoplasmic; Me3–malic enzyme; Pdhb–pyruvate dehydrogenase E1 component subunit beta.

**Figure 2:**
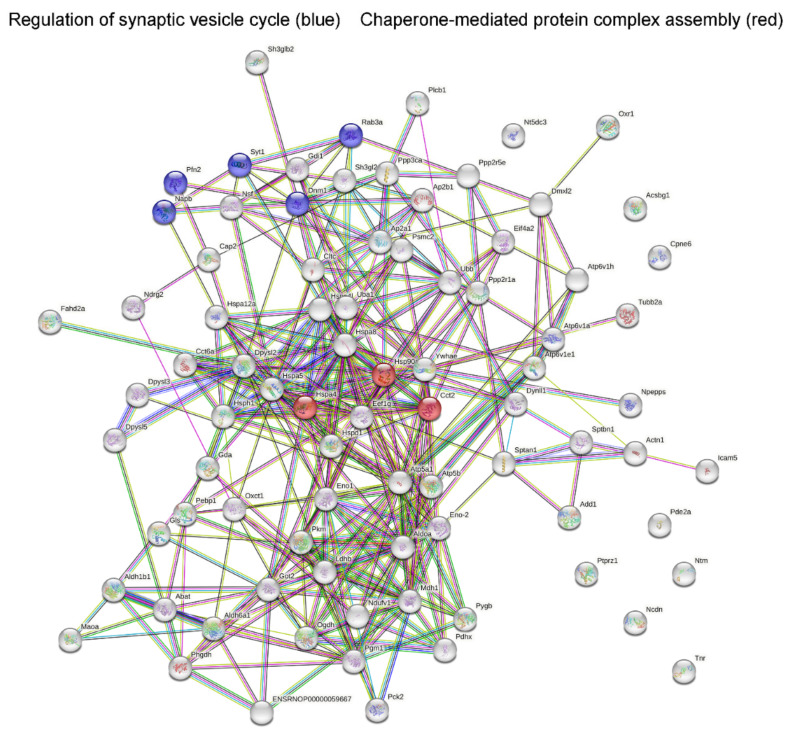
STRING-based detection of modified biological pathways among synaptosomal differentially upregulated proteins following Flx treatment in CSIS rats; in blue, proteins involved in regulation of synaptic vesicle cycle; in red, proteins involved in regulation of chaperone-mediated protein complex assembly; Napb–beta-soluble NSF attachment protein; Rab3a–Ras-related protein Rab-3A; Dnm1–dynamin-1; Syt1–synaptotagmin-1; Pfn2–profilin-2; Hsp90aa1–heat shock protein HSP 90-alpha; Hspa4–heat shock 70 kDa protein 4-like (predicted); Cct2–T-complex protein 1 subunit beta.

A schematic representation of a STRING-based interactome map of interactions among synaptosomal downregulated proteins in Controls treated to Flx with the most significant biological processes is presented in Figure 1. Synaptosomal upregulated proteins in Flx-treated CSIS rats with the most significant biological processes are shown in Figure 2.

### 2.3. Western Blot Analysis

**Figure 3:**
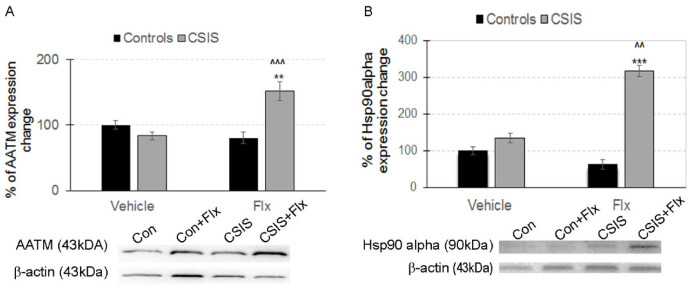
Validation of selected differentially expressed proteins in synaptosomal-enriched fractions of the rat hippocampus from Controls + Vehicle (Con), Controls + Fluoxetine (Con + Flx), Chronic Social Isolation + Vehicle (CSIS), and Chronic Social Isolation + Fluoxetine (CSIS + Flx) groups. Data are represented as % of protein expression change ± standard error of the mean (SEM), *n* = 3–6 rats per each group. Significant differences between groups obtained using a two-way ANOVA followed by Duncan’s post hoc test are indicated as follows: (**A**) aspartate aminotransferase, mitochondrial (AATM)–CSIS + Flx vs. CSIS (^^^^^ *p* < 0.001) and CSIS + Flx vs. Con (** *p* < 0.01); (**B**) heat shock protein 90 alpha (Hsp90 alpha)–CSIS + Flx vs. CSIS (^^^^ *p* < 0.01) and CSIS + Flx vs. Con (*** *p* < 0.001).

The expression differences of AATM and Hsp90 alpha (Figure 3A,B) were selected for Western blot validation of proteomics data as representatives of altered pathways in response to CSIS and/or Flx treatment. For ATTM expression, significant main effects of CSIS (F_1.19_ = 5.96, *p* < 0.05), Flx (F_1.19_ = 4.68, *p* < 0.05), and CSIS × Flx (F_1.19_ = 15.17, *p* < 0.001) were found, and upregulated expression in CSIS + Flx compared to CSIS (1.8 f.c., ^^^^^ *p* < 0.001) was confirmed by Western blot analysis (Figure 3A). For HSP 90 alpha expression, statistical analysis revealed a significant main effect of CSIS (F_1.10_ = 97.70, *p* < 0.001), Flx (F_1.11_ = 24.6, *p* < 0.001) and CSIS × Flx (F1.11 = 56.3, *p* < 0.001) with 2.4 f.c. (^* *p* < 0.01) upregulation in CSIS + Flx compared to CSIS (Figure 3B), which is in line with the liquid chromatography-tandem mass spectrometry (LC-MS/MS) results. Displayed blots are cropped images of representative examples of several Western blots performed. Full-length Western blot images are presented in Supplementary Figure S1.

## 3. Discussion

Synaptic dysfunction in MDD might be caused by the underlying changes in the expression of proteins. Hence, we profiled a nonhypothesis-driven comparative hippocampal synaptosomal proteome changes representative of the time-dependent expression changes underlying the development of CSIS-induced depressive-like behavior and Flx efficacy and identified affected signaling pathways and proteins following CSIS and/or Flx treatment.

Bioinformatics analysis of proteomic data demonstrated reduced expression of proteins in Flx-treated control rats (Table 1). Decreased expression levels of enzymes Eno1 (f.c. 0.78) and Aldoa (f.c. 0.75) involved in the glycolytic pathway, Mdh2 (f.c. 0.68) and Pdhb (f.c. 0.74) involved in TCA cycle, and two subunits composing ETC such as Atp5f1a (f.c. 0.80) and Cox6b1 (f.c. 0.50) were found. In support of these, decreased expression of CkB (f.c. 0.70), the energy storage enzyme which catalyzes the reversible exchange of high-energy phosphate, was revealed. Reduced expression of proteins involved in energy metabolism may reflect regulatory mechanisms in cells designed to limit unrestrained glucose consumption. We recently reported that Flx in the hippocampus of control rats stimulates energy metabolism by upregulating cytosolic GAPDH expression and directs energy metabolism toward the TCA and oxidative phosphorylation in nonsynaptic mitochondria (NSM) [16]. Differently obtained data may result in a different effect of Flx on the cell type of mitochondria (NSM vs. synaptic mitochondria). Flx treatment also altered the oxido-reduction process and downregulated protein expression of Gstp1 (f.c. 0.63) and Prdx3 (f.c. 0.65) involved in antioxidative defenses.

CSIS in control rats resulted in significant upregulation of synaptosomal proteins (Table 2). We found upregulated expression of Camk2a (f.c. 1.72), a protein that mediates intracellular signaling cascades and contributes to synaptic transmission as well as long-term potentiation (LTP) maintenance. Autophosphorylation of Camk2a is required for LTP and long-term memory [30]. Moreover, autophosphorylated Camk2a phosphorylates the AMPA-type glutamate receptor subunit GluA1, required for expression of LTP at mature hippocampal CA1 pyramidal cells [31,32]. In our study, increased expression of Camk2a may be associated with an attempt to overcome the stress condition.

CSIS also increased the expression of ATP8 (f.c. 5.77) (subunits of ATP synthase, Complex V) that catalyzes the formation of ATP from ADP and phosphate using the electrochemical gradient of protons across the inner mitochondrial membrane. This upregulation may increase ATP synthesis as a stress-coping mechanism in CSIS rats. Previous studies demonstrated that alterations in mitochondrial-mediated mechanisms might play a role in depression [21,33]. Nonetheless, we also found increased levels of Hspe1 (f.c. 2.1), chaperones involved in the processes of protein transport and assembly of multi-subunit protein complex that cause ATP consumption. Additionally, increased expression of polyubiquitin (f.c. 1.68) that serves as recognition signals for the proteasome was revealed. Contrary to this report, downregulated proteins involved in the ubiquitination process, as a part of the proteasome system, were found in the cytosol of the hippocampus of rats following CSIS. Moreover, differential expression patterns of the members of the ubiquitination family in response to CSIS probably depend on their subcellular compartments. However, given that expression of synaptosomal proteins following CSIS was increased, whereby the ubiquitin-proteasome pathway has a significant role in synaptic plasticity [34], it is likely to expect an increased participation/role of the chaperone/ubiquitous systems. 

By comparing Flx-treated CSIS and CSIS rats, synaptoproteome changes showed mainly upregulated protein expressions (Table 3). We found upregulated expression of proteins involved in vesicle-mediated transport in the synapse and synapse vesicle recycling (Dpysl2, Rab3a, Hspa8, Sh3gl2, Syt1 and Dnm1, f.c. 1.51–1.89). Moreover, the release of the neurotransmitters is primarily regulated by the presynaptic exo-endocytic cycle [35]. Hence, several proteins involved in synaptic vesicle exocytosis (Rab3a, Nsf, Syt1, and Ppp3ca, f.c. 1.52–2.15) and endocytosis (Hspa8, Sh3gl2, Syt1, Dnm1, f.c. 1.57–1.89) were upregulated. Synaptotagmin-1 (Syt1, f.c. 1.85), a synaptic vesicle protein, serves as a dual Ca^2+^ sensor for exocytosis and endocytosis [36]. It triggers vesicle release [37] along with Rab (f,c, 1.52) proteins involved in vesicle docking [38] and Nsf (1.57) that regulates the neurotransmitter release and maintains the readily releasable pool of synaptic vesicles [39]. Our data are corroborated by a study demonstrating that chronic Flx treatment is associated with increased expression of proteins related to vesicular trafficking and release, such as Syt1 [15]. Nonetheless, Ap2a1 and Ap2b1 (f.c. 1.76–2.20), as parts of the AP-2 complex involved in the recycling process of synaptic vesicles, along with Clct (f.c. 1.88) that forms clathrin-coated vesicles at the plasma membrane [40] and Dnm1 (f.c. 1.89), implicated in endocytotic synaptic vesicles fission at the presynaptic plasma membrane [41], were upregulated. This is supported by upregulated endophilins (Sh3gl2, Sh3glb2, f.c. 1.69–1.72) as a component of clathrin-mediated endocytosis [42]. Taken together, Flx-induced increase in expression of synaptic proteins involved in exo/endocytosis in the CSIS rats might reflect a dynamic response to changes in synaptic stimulation, allowing neurons to maintain the necessary level of neurotransmitters and resembles the effect of common antidepressant action. 

Flx in CSIS rats resulted in increased expression of proteins involved in synaptic mitochondria bioenergetic pathways. Several of these proteins are involved in TCA cycle (Mdh1, Ogdh, f.c. 1.53–1.69), ETC Complexes I (Ndufv1, f.c. 1.60), and V (Atp5f1a, Atp5f1b, f.c. 2.03–2.21), indicating the Flx-induced increase in synaptic mitochondrial energy production is a protective effect. Upregulated protein expression of V-type proton (H^+^) ATPase (V-ATPase) (Atp6v1a, Atp6v1e1, Atp6v1h, f.c. 1.55–2.20), which generates a proton gradient across the vesicular membrane, likely increases active transport of neurotransmitters into synaptic vesicles and synaptic transmission [43,44]. However, our results showed downregulated proteins of two subunits, such as Ndufa2 (complex I, f.c. 0.73) and Atp5me (Complex V, f.c. 0.36). Regardless of how expression changes occur, the effect of Flx on synaptic energy metabolism will depend on enzyme activities.

Nonetheless, the levels of Mdh1 (f.c. 1.53) and aspartate aminotransferase, mitochondrial (AATM or Got2 (f.c. 1.72)) involved in the malate-aspartate shuttle were increased, whereby expression change of AATM, validated by Western blot analysis (1.8 f.c.) (Figure 3A), is in accordance with proteomic data. This shuttle promotes transport of cytosolic NADH into the mitochondria, whereby regulation of NAD^+^/NADH ratio aids oxidative metabolism of glucose and synthesis of neurotransmitter glutamate from glutamine in the brain [45]. Interestingly, we found increased expression of Kyat3 (f.c.1.86) enzyme that catalyzes the irreversible transamination of L-kynurenine, a product of tryptophan metabolism, to the neuroprotective glutamate receptor antagonist kynurenic acid (KYNA). In support of this, AATM (f.c. 1.72) also plays a role in KYNA formation [46]. Moreover, future studies will examine the targeting possible pathways responsible for these mechanisms of action.

Importantly, proteins involved in intracellular protein transport (Hsp90aa1, Gdi1, Ywhae, Pde2a, f.c. 1.56–1.82) and chaperone-mediated protein transport (Hspd1, Hsp90aa1, Hspa8, f.c. 1.54–1.57) were upregulated. Regarding Hsp90aa1, Western blot analysis confirmed increased expression of proteomic data (2.4 f.c.) (Figure 3B). Chaperones are required for proper protein folding and transport, consuming ATP in their function [47]. This result might be an adaptive change of the cell attempting to assemble individual proteins into functional complexes. In contrast, HSP10, a chaperone with the same role, was downregulated. Due to the disordered nature of unfolded or aggregated proteins, most probably different chaperones will be included in targeting misfolded proteins. 

Comparing the results between CSIS vs. Controls and CSIS + Flx vs. CSIS, only two proteins, Ppp2r1a and Hspe1, were upregulated by CSIS (Supplementary Table S1). The reason could lie in altered homeostasis of CSIS rats, causing different sensitivity of cells to the treatment. Comparing Flx-treated controls with Flx-treated CSIS, Flx additionally stimulated the expression of four proteins in CSIS rats (Supplementary Table S2) involved in energy processes.

### Limitation

There are two limitations to the current study. First, proteomic data obtained from LC-MS/MS analysis were derived from a single determination performed on one pooled sample of each experimental group, and therefore appropriate statistical analysis was not performed. Even though sample pooling does not provide a characterization of biological variance, the pooling approach is a preferable approach to reliably indicate a common pattern in the expression of proteins [48]. Second, although LC-MS/MS analysis includes the entire hippocampus, an anatomically defined brain area, it will be important to examine its dorsal and ventral part which may play significantly different roles in cognitive functions, and also likely in MDD. Therefore, any results we gain from molecular studies such as this cannot be readily applied as a “function” of the “entire” hippocampus.

## 4. Materials and Methods

### 4.1. Animals

The Animal Facility of “VINČA” Institute of Nuclear Sciences–National Institute of thе Republic of Serbia, University of Belgrade, provided adult male Wistar rats (2 months old, 200–300 g weight). Rats were housed under standard conditions in groups of four per cage on a 12 h/12 h light/dark cycle (lights on between 07:00 and 19:00 h), in a temperature-controlled environment (20 ± 2 °C), and humidity 55 ± 10%, with access to water and food (commercial rat pellets) ad libitum. Rats were monitored daily. All experimental procedures are reported following the recommendations of the ARRIVE guidelines.

### 4.2. Study Design

**Figure 4:**
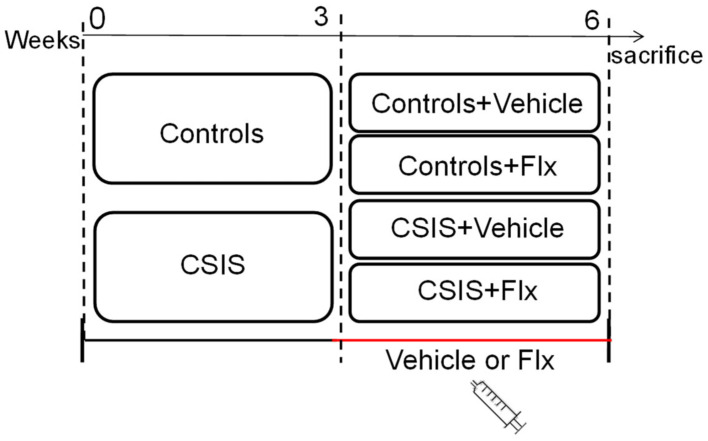
Schematic representation of the study design. CSIS–Chronic Social Isolation; Flx-fluoxetine.

At the beginning of the experiment, rats were randomly divided into two separate batches, with half of the animals being control rats (housed in groups of up to four), while the other half of the animals were exposed to CSIS (rats housed individually in cages, deprived of any tactile or visual contact but with normal olfactory and auditory experiences). The experiment contained two parts with a total duration of 6 weeks (Figure 4). During the first 3 weeks of the experiment, rats were housed under the aforementioned conditions without additional experimental procedures. During the second 3 weeks, half of Controls and CSIS rats were intraperitoneal (i.p) treated with Flx solution (15 mg/kg/day) (Controls + Flx and CSIS + Flx), while the rest were i.p. treated with physiological solution (Controls + Vehicle and CSIS + Vehicle). Depressive- and anxiety-like behaviors in rats were determined according to a significant decrease in sucrose preference [49], an increase in buried marbles [50], and increased immobility in the forced swim test [51], as previously published [23,24,25]. 

### 4.3. Fluoxetine-Hydrochloride Administration

Flunisan tablets (containing 20 mg of fluoxetine-hydrochloride, Hemofarm AD Vršac, Vršac, Serbia) were used to prepare fluoxetine-hydrochloride (hereafter referred to as Flx) solution for the treatment. Tablets were crushed and the content dissolved in distilled, sterile water. The resulting suspension was mixed on a magnetic stirrer and filtered through Whatman No. 42 filter paper. Ultra Performance Liquid Chromatography analysis was used to determine the concentration of Flx solution. Flx was administered (15 mg/kg/day) according to rats’ body weights measured weekly. Treatment with Flx for 3 weeks resulted in a serum concentration of 280 ± 50 ng/mL in Flx-treated controls and 230 ± 28 ng/mL in Flx-treated CSIS animals, as measured 24 h after the last treatment (Perić et al., 2017); these levels corresponded to those reported in the serum of patients after treatment with doses of 20–80 mg/day of Prozac (100–700 ng/mL) [52]. 

### 4.4. Preparation of Synaptosomal Fractions from the Rat Hippocampus

Twenty-four hours after the end of behavior experiments, rats were anesthetized with a mixture of ketamine/xylazine (100/5 mg/kg), intracardially perfused with ice-cold physiological saline up to 50 mL of volume and sacrificed by guillotine decapitation. Brains were removed quickly, kept on an ice-cold plate immediately, and hippocampi were dissected. To obtain synaptosomal fractions, both hippocampal hemispheres from three rats in each group were pooled in one sample, whereby each group contained three samples, i.e., control (*n* = 3), control + Flx (*n* = 3), CSIS (*n* = 3) and CSIS + Flx (*n* = 3), to ensure sufficient amounts of tissue for the synaptosomal fraction. The final number of individual hippocampi included in data analysis per group was *n* = 9. Deep-frozen rat hippocampi were homogenized with Potter-Elvehjem glass homogenizer with a teflon pestle (10 up-and-down even strokes, 800 rpm) in cold homogenization buffer (0.25 M sucrose (Fisher Scientific), 10 mM Tris/HCl (SERVA) pH 7.4, containing protease inhibitor cocktail tablet (complete tablets, Mini, EASY pack, Roche)). Homogenates were centrifuged at 1300× *g* for 10 min at 4 °C for removing the pellet of nuclei, followed by further re-centrifugated to remove the remaining nuclei under the same conditions. Obtained supernatants were centrifuged on 19,200× *g* at 4 °C for 15 min to obtain the crude mitochondrial pellets, containing synaptosomes. The resulting supernatants were centrifuged on 100,000× *g* at 4 °C for 45 min to obtain pure cytosolic fractions. Percoll (GE Healthcare) discontinuous density gradient (15%, 24%, and 40%) in sucrose buffer was used for separation of NSM from synaptosomal fraction [53]. The synaptosomal fractions were collected at 14/24% interface, while the NSM were collected at 24/40% interface, after centrifugation for 15 min at 37,000× *g*, at 4 °C. Synaptosomal fractions were cleaned two times in ten volumes of homogenization buffer, centrifuged at 14,000× *g*, at 4 °C for 30 min and obtained pellets resuspended in lysis buffer (5 mM Tris-HCl pH 8.1, 0.5 mM EDTA). All fractions were stored at −80 °C until further analyses. The protein concentrations were estimated in all samples by method of Lowry (1951) [54]. Purified bovine serum albumin (BSA) was used as a standard. The relative purity of isolated subcellular fractions by selected protocol was verified using specific protein markers of cellular components of the control sample. The major distribution of TATA binding protein, α tubulin and synaptophysin in nuclear, cytosolic, and synaptosomal fractions, respectively, indicated that these fractions were relatively free of contamination, as demonstrated in our previous study [24].

### 4.5. Electrophoresis, In-Gel Digestion and LC-MS/MS

The synaptosomal fractions of all four groups were separated by SDS polyacrylamide gel electrophoresis (SDS-PAGE) using NuPAGE 4–12% Bis-Tris Gels (Life Technologies, Carlsbad, CA, USA), followed by in-gel digestion, as previously described [24]. The analysis was performed with HPLC-LTQ Orbitrap XL mass spectrometer [24]. The Uniprot/Swissprot database using the Proteome Discoverer browser (version 1.3) (Thermo Fisher Scientific, Waltham, MA, USA), was used to search the extracted MS/MS spectra, whereby common variable modifications and one missed tryptic cleavage were accepted. Peptide tolerance was ±10 ppm, and MS/MS tolerance was ±0.5 Da. All protein identification experiments were performed using the corresponding decoy database and a false discovery rate (FDR) of 1%. The label-free quantification (LFQ) tool of the Sieve 2.0 software (Thermo Fisher Scientific) using a mass error tolerance of ±10 ppm and a retention time shift of ±1 min was used for the relative quantification of the proteins. Proteins identified with at least two peptides match and/or unique peptide were considered for relative quantification. Bioinformatic analysis was performed for every up/downregulated protein according to their UniProtKB accession numbers using STRING (version 11.0). The mass spectrometry proteomics row data have been deposited to the ProteomeXchangeConsortium via the PRIDE [55] partner repository with the dataset identifier PXD028816.

### 4.6. Western Blot Validation of the Proteomic Results 

Western blot was used for validation of the proteomic data for selected proteins on a separate batch of animals (*n* = 3–6 per group) that underwent the same procedure as explained in the study design. Briefly, equal amounts of protein samples (6 µg) were loaded on a 10% SDS-PAGE for separation followed by transfer onto a polyvinylidene difluoride membrane. The membrane was kept in 5% BSA Fraction V (Sigma, A9418, St. Louis, MO, USA) containing Tris-buffered saline (TBS), pH 7.5 for 1h at room temperature (RT) and then incubated overnight at 4 °C with primary antibodies diluted in TBS. Prior to the hybridization with primary antibodies, membranes were cut at a desirable range of protein mass (kDa) based on the Thermo Scientific PageRuler Plus Prestained Protein Ladder (#26619). We used antiaspartate aminotransferase, mitochondrial (AATM, Santa Cruz, sc-271702, 1/1000) (Molecular weight (Mw) of 43 kDa, detected between 35–70 kDa) and anti-HSP 90α/β (Santa Cruz, sc-13119, 1:1000) (Mw of 90 kDa, detected between 70–130 kDa) and anti-β actin (Santa Cruz, sc-47778, 1:1000) (Mw of 43 kDa, detected between 35–70 kDa) as a loading control followed by 1 h incubation at RT with secondary anti-mouse (A9917, Sigma Aldrich, 1:10,000) antibody conjugated with horseradish peroxidase. Immobilon Western chemiluminescent HRP substrate (Millipore, Burlington, MA, USA) was used to induce the chemiluminescent signal, and relative optical density of protein bands was detected with the Chemidoc-MP System (Bio-Rad, Hercules, CA, USA). Quantitative analysis of protein band was conducted by Image Lab 5.0 software (Bio-Rad). After imaging of AATM, the membranes were stripped using a mild stripping buffer (0.015% *w*/*w* glycine, 0.001% *w*/*w* SDS, 0.010% *v*/*w* Tween 20, pH 2.2) to remove chemiluminescent HRP substrate and previously bound antibody while preserving protein content. After 20 min incubation (2 × 10 min) with the stripping buffer, the membranes were washed and blocked in a 5% BSA Fraction V and reprobed with β-actin (Santa Cruz, sc-47778, 1:1000) overnight at 4 °C.

### 4.7. Bioinformatics and Statistical Analysis

The interactome network analysis was conducted by STRING (version 11), complemented with a biological process, molecular functions, and KEGG pathways. Proteomic data are presented according to software pre-set at *p* < 0.01 for peptides and *p* < 0.05 for proteins. Proteins with a fold change (f.c.) greater than or equal to 1.5 (f.c. ≥ 1.5) or less or equal to 0.80 (f.c. ≤ 0.80) were considered differentially expressed. Western blot data showed normal distribution according to the Shapiro–Wilk test and equal variances by Levene’s test. Then, a two-way ANOVA was performed (the factors were treatment (levels: vehicle and Flx) and condition (controls and CSIS), followed by Duncan’s post hoc test, using Statistica 10. The number of animals per group was *n* = 3–6. Statistical significance was set at *p* < 0.05. All data are expressed as the mean ± SEM.

## 5. Conclusions

The results showed that Flx treatment of control rats induced downregulation of proteins involved in mitochondrial energy processes (TCA cycle, ETC) and redox system enzymes. A comparison of CSIS and control rats showed upregulation of synaptosomal protein expression mainly participating in Camk2-related neurotransmission, vesicle transport, ubiquitination, and mitochondrial energy processes. Flx application to CSIS rats predominantly increases vesicle trafficking and mitochondrial bioenergetics, which might be the potential targets for therapeutic treatments in MDD. The identified synaptic and mitochondrial proteins and altered molecular pathways suggest them as potential synaptic markers and targets for Flx treatments and potentially crucial for the effective treatment of stress-related MDD.

## Figures and Tables

**Table 1:** Synaptosomal differentially downregulated expressed proteins in Controls + Fluoxetine vs. Controls.

Name	Accession No	Gene	Ratio	Matched Peptides	Unique Peptides
ATP synthase subunit alpha, mitochondrial	P15999	*Atp5f1a*	0.80	11	10
Malic enzyme	A0A0G2K4C6	*Me3*	0.79	2	2
RAB5B, member RAS oncogene family	A1L1J8	*Rab5b*	0.79	3	2
Enolase 1, (Alpha)	Q5EB49	*Eno1*	0.78	18	16
Annexin	O70371	N/A	0.77	15	15
Septin 5	D3ZDH8	*Sep5*	0.77	5	5
Malate dehydrogenase, cytoplasmic	O88989	*Mdh1*	0.76	13	13
F-actin-capping protein subunit alpha-2	Q3T1K5	*Capza2*	0.76	3	3
Fructose-bisphosphate aldolase A	P05065	*Aldoa*	0.75	22	18
NAD-dependent protein deacetylase sirtuin-2	Q5RJQ4	*Sirt2*	0.75	3	3
Pyruvate dehydrogenase E1 component subunit beta	A0A0G2KAM3	*Pdhb*	0.74	18	16
Cofilin-1	P45592	*Cfl1*	0.74	2	2
Annexin	Q5U362	*Anxa4*	0.72	6	6
Creatine kinase B-type	P07335	*Ckb*	0.70	27	25
Annexin	Q6IMZ3	*Anxa6*	0.70	17	15
Malate dehydrogenase, mitochondrial	P04636	*Mdh2*	0.68	31	30
Peroxiredoxin 3	G3V7I0	*Prdx3*	0.65	3	3
Glutathione S-transferase P	P04906	*Gstp1*	0.63	4	4
Annexin A3	P14669	*Anxa3*	0.62	11	11
Cytochrome c oxidase subunit	D3ZD09	*Cox6b1*	0.50	2	2

**Table 2:** Synaptosomal differentially upregulated expressed proteins in chronic social isolation (CSIS) vs. Controls.

Name	Accession No	Gene	Ratio	Matched Peptides	Unique Peptides
ATP synthase protein 8	Q5UAJ5	*ATP8*	5.77	2	2
Protein phosphatase 2 (Formerly 2A), regulatory subunit A (PR 65), alpha isoform, isoform CRA_a	Q5XI34	*Ppp2r1a*	2.25	2	2
Tropomyosin alpha-3 chain	Q63610	*Tpm3*	2.11	5	3
10 kDa heat shock protein, mitochondrial	P26772	*Hspe1*	2.10	8	8
Calcium/calmodulin-dependent protein kinase type II subunit alpha	P11275	*Camk2a*	1.72	5	3
Polyubiquitin	Q63654	*UBC*	1.68	6	2
Isoform Non-brain of Clathrin light chain	P08082-2	*Cltb*	1.64	2	2
Elongation factor 1-gamma	Q68FR6	*Eef1g*	1.52	3	3

**Table 3:** Synaptosomal differentially expressed proteins in fluoxetine-treated chronically socially isolated rats (CSIS + Flx) vs. CSIS.

Name	Accession No	Gene	Ratio	Matched Peptides	Unique Peptides
Serine/threonine-protein phosphatase 2A 56 kDa regulatory subunit	A0A0G2JTA1	*Ppp2r5e*	2.31	3	2
Tubulin beta-2A chain	P85108	*Tubb2a*	2.27	15	5
ATP synthase subunit beta	G3V6D3	*Atp5f1b*	2.21	26	24
V-type proton ATPase subunit H	A0A0G2K9J2	*Atp6v1h*	2.20	6	5
AP-2 complex subunit beta	P62944	*Ap2b1*	2.20	11	5
T-complex protein 1 subunit beta	Q5XIM9	*Cct2*	2.17	3	3
Serine/threonine-protein phosphatase 2B catalytic subunit alpha isoform	P63329	*Ppp3ca*	2.15	8	6
Alpha-actinin-1	Q9Z1P2	*Actn1*	2.03	14	8
ATP synthase subunit alpha, mitochondrial	P15999	*Atp5f1a*	2.03	14	14
26S proteasome regulatory subunit 7	Q63347	*Psmc2*	2.00	2	2
Beta-soluble NSF attachment protein	F8WFM2	*Napb*	1.97	7	6
Neurochondrin	O35095	*Ncdn*	1.92	8	6
Long-chain-fatty-acid--CoA ligase ACSBG1	Q924N5	*Acsbg1*	1.89	5	3
Dynamin-1	P21575	*Dnm1*	1.89	32	29
Clathrin heavy chain 1	P11442	*Cltc*	1.88	75	71
Clathrin heavy chain	F1M779	*Cltc*	1.88	75	72
Phosphoglucomutase 1	Q499Q4	*Pgm1*	1.87	5	5
4-aminobutyrate aminotransferase, mitochondrial	P50554	*Abat*	1.87	17	16
Heat shock 70kDa protein 12A (Predicted), isoform CRA_a	D3ZC55	*Hspa12a*	1.86	3	3
Kynurenine--oxoglutarate transaminase 3	Q58FK9	*Kyat3*	1.86	3	3
Adducin 1 (Alpha), isoform CRA_b	A0A0G2JSM7	*Add1*	1.86	5	4
Synaptotagmin-1	P21707	*Syt1*	1.85	4	3
Dihydropyrimidinase-related protein	Q9JMG8	N/A	1.83	3	3
Glutaminase kidney isoform, mitochondrial	P13264	*Gls*	1.83	14	10
Dmx-like 2	F1M3W5	*Dmxl2*	1.82	2	2
Phosphodiesterase	F8WFW5	*Pde2a*	1.82	2	2
Fumarylacetoacetate hydrolase domain-containing protein 2	B2RYW9	*Fahd2*	1.79	5	5
Polyubiquitin-C	F1LML2	*Ubc*	1.79	7	6
Guanine deaminase	Q9JKB7	*Gda*	1.79	11	11
Copine 6 protein	H1UBM5	*Cpne6*	1.78	6	5
Heat shock protein 105 kDa	Q66HA8	*Hsph1*	1.78	12	8
Aspartate aminotransferase, mitochondrial	P00507	*Got2*	1.76	5	2
AP-2 complex subunit alpha	D3ZUY8	*Ap2a1*	1.76	14	7
Endophilin-B2	Q5PPJ9	*Sh3glb2*	1.72	3	2
Eukaryotic initiation factor 4A-II	Q5RKI1	*Eif4a2*	1.72	4	4
Chaperonin containing Tcp1, subunit 6A (Zeta 1)	Q3MHS9	*Cct6a*	1.70	3	2
Gamma-enolase	P07323	*Eno2*	1.69	10	7
2-oxoglutarate dehydrogenase, mitochondrial	Q5XI78	*Ogdh*	1.69	12	8
Endophilin-A1	O35179	*Sh3gl2*	1.69	11	10
Rab GDP dissociation inhibitor alpha	P50398	*Gdi1*	1.67	29	21
Adenylyl cyclase-associated protein 2	P52481	*Cap2*	1.67	7	5
Aldehyde dehydrogenase X, mitochondrial	G3V7I5	*Aldh1b1*	1.66	7	5
Oxidation resistance protein 1	A0A0G2K7Y2	*Oxr1*	1.64	6	5
Protein NDRG2	Q8VBU2	*Ndrg2*	1.64	9	9
Spectrin beta chain	A0A0G2K8W9	*Sptbn1*	1.64	23	19
Amine oxidase	B2GV33	*Maoa*	1.63	3	3
1-phosphatidylinositol 4,5-bisphosphate phosphodiesterase beta-1	P10687	*Plcb1*	1.62	2	2
ATPase, H+ transporting, V1 subunit E isoform 1, isoform CRA_a	G3V7L8	*Atp6v1e1*	1.62	9	8
Endoplasmic reticulum chaperone BiP	P06761	*Hspa5*	1.62	13	10
Pyruvate kinase PKM	P11980	*Pkm*	1.61	34	4
NADH dehydrogenase [ubiquinone] flavoprotein 1, mitochondrial	Q5XIH3	*Ndufv1*	1.60	4	4
Pck2 protein	B2RYG2	*Pck2*	1.60	12	12
Ubiquitin-like modifier-activating enzyme 1	Q5U300	*Uba1*	1.60	16	14
14-3-3 protein epsilon	P62260	*Ywhae*	1.60	18	15
Heat shock 70kDa protein 4-like (Predicted), isoform CRA_b	B4F772	*Hspa4l*	1.59	9	7
Dihydropyrimidinase-related protein 3	Q62952	*Dpysl3*	1.58	10	5
Dihydrolipoamide acetyltransferase component of pyruvate dehydrogenase complex	A0A0G2JZH8	*Pdhx*	1.58	9	7
Alpha-1,4 glucan phosphorylase	G3V6Y6	*Pygb*	1.58	12	8
Protein phosphatase 2 (Formerly 2A), regulatory subunit A (PR 65), alpha isoform, isoform CRA_a	Q5XI34	*Ppp2r1a*	1.58	10	10
D-3-phosphoglycerate dehydrogenase	O08651	*Phgdh*	1.57	4	3
N-ethylmaleimide sensitive fusion protein, isoform CRA_b	F1LQ81	*Nsf*	1.57	22	21
Heat shock cognate 71 kDa protein	P63018	*Hspa8*	1.57	44	36
Heat shock 70 kDa protein 4	F1LRV4	*Hspa4*	1.56	16	12
Heat shock protein HSP 90-alpha	P82995	*Hsp90aa1*	1.56	27	20
Dynein light chain 1, cytoplasmic	P63170	*Dynll1*	1.55	2	2
Elongation factor 1-gamma	Q68FR6	*Eef1g*	1.55	2	2
Methylmalonate-semialdehyde dehydrogenase [acylating], mitochondrial	Q02253	*Aldh6a1*	1.55	3	3
Tenascin R, isoform CRA_b	A0A096MJE6	*Tnr*	1.55	12	10
ATPase H^+^-transporting V1 subunit A	D4A133	*Atp6v1a*	1.55	39	38
5′-nucleotidase domain-containing 3	D3ZAI6	*Nt5dc3*	1.54	8	7
Aminopeptidase	F1M9V7	*Npepps*	1.54	8	7
L-lactate dehydrogenase B chain	P42123	*Ldhb*	1.54	15	12
60 kDa heat shock protein, mitochondrial	P63039	*Hspd1*	1.54	39	36
Phosphatidylethanolamine-binding protein 1	P31044	*Pebp1*	1.53	3	3
Malate dehydrogenase, cytoplasmic	O88989	*Mdh1*	1.53	12	12
Intercellular adhesion molecule 5	D4A435	*Icam5*	1.52	4	4
Fructose-bisphosphate aldolase A	P05065	*Aldoa*	1.52	5	4
Ras-related protein Rab-3A	P63012	*Rab3a*	1.52	11	10
Spectrin alpha chain, nonerythrocytic 1	A0A0G2JZ69	*Sptan1*	1.52	44	35
Receptor-type tyrosine-protein phosphatase zeta	F1LMY3	*Ptprz1*	1.51	2	2
Opioid-binding protein/cell adhesion molecule	P32736	*Opcml*	1.51	4	3
Succinyl-CoA:3-ketoacid coenzyme A transferase 1, mitochondrial	B2GV06	*Oxct1*	1.51	16	15
Enolase 1, (Alpha)	Q5EB49	*Eno1*	1.51	22	19
Dihydropyrimidinase-related protein 2	P47942	*Dpysl2*	1.51	49	41
Profilin-2	Q9EPC6	*Pfn2*	1.50	2	2
Microtubule-associated protein 6	Q63560	*Map6*	0.79	3	3
10 kDa heat shock protein, mitochondrial	P26772	*Hspe1*	0.79	6	6
NADH dehydrogenase [ubiquinone] 1 alpha subcomplex subunit 2	D3ZS58	*Ndufa2*	0.73	2	2
Isoform Excitatory amino acid transporter 2	P31596-2	*Slc1a2*	0.73	7	7
Myelin proteolipid protein	P60203	*Plp1*	0.69	6	5
ATP synthase subunit e, mitochondrial	P29419	*Atp5me*	0.36	4	3

## Data Availability

Proteomics data is available via ProteomeXchangeConsortium via the PRIDE partner repository with the dataset identifier PXD028816.

## Supplementary Materials

The following supporting information can be downloaded at: https://www.mdpi.com/article/10.3390/ijms232315351/s1, Table S1. Overlap of differentially expressed proteins between Flx-treated CSIS rats and following CSIS exposure; Table S2. Overlap of differentially expressed proteins between Flx-treated CSIS rats and Flx-treated Control rats; Figure S1. Full-length Western blot (WB) images represented in Figure 4 in controls + vehicle (Con), fluoxetine-treated controls (Con + Flx), chronic social isolation (CSIS) and fluoxetine-treated CSIS (CSIS + Flx) rats.
