# Supplementary figures and images for: Fluoxetine Enhances Synaptic Vesicle Trafficking and Energy Metabolism in the Hippocampus of Socially Isolated Rats
Source: Int J Mol Sci. 2022 Dec 5;23(23):15351. doi: 10.3390/ijms232315351 (PMC9735484; doi:10.3390/ijms232315351)

**WB AATM (Aspartate Aminotransferase Mitochondrial)/β actin**

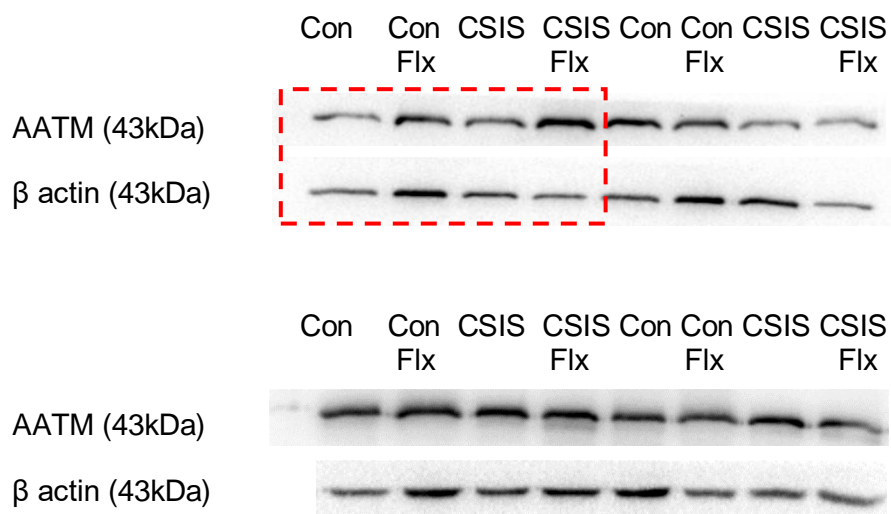

Supplement: Supplementary file 1 [file ijms-23-15351-s001.zip › Suplementary Figure S1.pdf]
